# Accuracy and sensitivity of four‐dimensional dose calculation to systematic motion variability in stereotatic body radiotherapy (SBRT) for lung cancer

**DOI:** 10.1120/jacmp.v13i6.3992

**Published:** 2012-11-08

**Authors:** Mark K.H. Chan, Dora L.W. Kwong, Sherry C.Y. Ng, Anthony S.M. Tong, Eric K.W. Tam

**Affiliations:** ^1^ Department of Clinical Oncology Tuen Mun Hospital Hong Kong (S.A.R); ^2^ Department of Clinical Oncology The University of Hong Kong Hong Kong (S.A.R); ^3^ Department of Clinical Oncology Queen Mary Hospital Hong Kong (S.A.R); ^4^ Theresa Po Cyberknife Center Hong Kong (S.A.R)

**Keywords:** 4D dose calculation, CyberKnife, SBRT

## Abstract

The dynamic movement of radiation beam in real‐time tumor tracking may cause overdosing to critical organs surrounding the target. The primary objective of this study was to verify the accuracy of the 4D planning module incorporated in CyberKnife treatment planning system. The secondary objective was to evaluate the error that may occur in the case of a systematic change of motion pattern. Measurements were made using a rigid thorax phantom. Target motion was simulated with two waveforms (sin and ${\text{cos}}^{4}$) of different amplitude and frequency. Inversely optimized dose distributions were calculated in the CyberKnife treatment planning system using the 4D Monte Carlo dose calculation algorithm. Each plan was delivered to the phantom assuming (1) reproducible target motion, and (2) systematic change of target motion pattern. The accuracy of 4D dose calculation algorithm was assessed using GAFCHROMIC EBT2 films based on 5%/3 mm γ criteria. Treatment plans were considered acceptable if the percentage of pixels passing the 5%/3 mm γ criteria was greater than 90%. The mean percentages of pixels passing were 95% for the target and 91% for the static off‐target structure, respectively, with reproducible target motion. When systematic changes of the motion pattern were introduced during treatment delivery, the mean percentages of pixels passing decreased significantly in the off‐target films (48%; p < 0.05), but did not change significantly in the target films (92%; p=0.324) compared to results of reproducible target motion. These results suggest that the accuracy of 4D dose calculation, particularly in off‐target stationary structure, is strongly tied to the reproducibility of target motion and that the solutions of 4D planning do not reflect the clinical nature of nonreproducible target motion generally.

PACS numbers: 87.53.Ly, 87.55.km

## I. INTRODUCTION

Real‐time tumor tracking (RTRT) has been increasingly employed in stereotactic body radiotherapy (SBRT).^1^, ^2^ Through continuous adaptation of radiation beam to the moving target, RTRT can effectively eliminate the systematic and random errors due to target motion and thus allow great potential of dose escalation by significant margin reduction.^(3)^ CyberKnife is one of the few RTRT systems that are presently applied to clinical SBRT treatments. Tumor tracking by CyberKnife is implemented under two subsystems of the target localization system (TLS) called Synchrony Respiratory Tracking System (RTS) and the XSight Lung Tracking System. Both tracking systems involve a correspondence model that combines continuously acquired respiratory signal and continually updated kV stereoscopic image pairs of internal fiducial markers (i.e., Synchrony RTS) or soft tumor tissue (i.e., XSight Lung Tracing System). During treatment, these tracking systems measure the external breathing signal to predict the tumor position based on the correspondence model and drive the robotic arm to track the rigid target movement accordingly.

One major concern of these beam tracking treatments is that standard treatment plans are created on static anatomy and are subsequently delivered to dynamically changing anatomy. That is, treatment plans of 3D nature are delivered in 4D manner. Because internal organ motion is nonrigid, the positions of the target and hence the radiation beam relative to other critical organs, such as esophagus and spinal cord, can change during respiration. A few studies have demonstrated that failure to account for the organ deformation and the dynamic tracking beam movement in the treatment planning process may cause unexpected loss of target dose coverage and increase of critical organ doses.^4^, ^5^ To resolve this problem, a 4D dose optimization and calculation algorithm has been added to the CyberKnife treatment planning system (TPS). Similar to other 4D dose calculation methods,^6^, ^7^ this algorithm takes 4D CT as input to provide the breathing geometry and determine the target trajectory, and uses deformation image registration (DIR) as a tool to deform the dose distribution of one breathing geometry to the others. Theoretically, 4D dose calculation should have the potential to minimize the errors between delivered and planned dose distributions.^(8)^ However, several studies have shown that interpolation errors of the DIR and dose calculation may produce inaccurate dose distributions.^9^, ^10^ Furthermore, 4D dose calculation shares the same assumption with conventional 3D dose calculations that the breathing motion during imaging and treatment is reproducible. Unfortunately, this assumption does not hold in most clinical situations. Different studies have previously demonstrated that tumors may change in their motion trajectory, amplitude, frequency, or baselines on different time scale, causing intrafractional and interfractional variations of the patient model.^1^, ^11^ 4D treatment planning based on a single patient breathing model can introduce systematic error that cannot be simply averaged out by multiple treatment deliveries, as has been discussed by Evans et al.^(12)^ This work aimed to use a commercial dynamic thorax phantom to investigate the accuracy of 4D CyberKnife treatment plans and also to evaluate the validity of 4D calculation in the presence of a systematic change in motion pattern.

## II. MATERIALS AND METHODS

**Figure 1:**
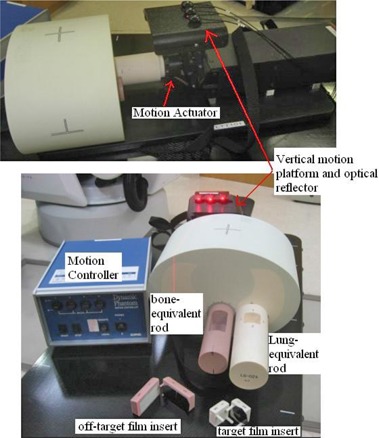
Components of the dynamic thorax phantom.

Figure 1 shows the dynamic thorax phantom (Model 18043, CIRS Inc., Norfolk, VA used in this study. The thorax body houses two cylinder rods made of lung‐ and trabecular bone‐equivalent materials, respectively. The lung‐equivalent rod has a target film cube with a 25 mm (diameter) simulated tissue tumor sphere that accommodates two orthogonal radiochromic films. The bone‐equivalent rod also contains a cylindrical critical structure that accommodates film in the coronal plane. The dynamic phantom has a motion controller connected to a motion actuator to produce three‐dimensional translation and rotation motion of the lung‐equivalent rod. To facilitate phantom setup, there are four metal fiducials embedded in the phantom, two affixed to the target and the other affixed to the cord cylinder. In addition, the phantom has an independent motion platform providing the external breathing signal needed to build the correspondence model of Synchrony RTSTM (CyberKnife; Accuray Inc., Sunnyvale, CA).

The simulated target was programmed to move in seven different patterns (Table 1). Both simple one‐dimensional (1D) and 3D motions were included. The choice of motion period T and amplitude A were based on the analysis of patients treated at our institute, who showed an averaged 3D motion of 8.6 mm and period of four seconds. A large 30 mm superior–inferior (SI) motion was also included as an extreme scenario. The influence of asymmetric motion was investigated using a sin waveform and a ${\text{cos}}^{4}$ waveform. The ${\text{cos}}^{4}$ function has been demonstrated to provide the best fit to the population data.^(13)^

**Table 1:** Summary of the programmed target motion.

		*Motion Parameters*			
*Motion Pattern*	*Superior–Inferior (mm)*	*Anterior–Posterior (mm)*	*Left–Right (mm)*	*Period (s)*	*Waveform*
M_1	10	5	2	4	${\text{cos}}^{4}$
M_2	20	5	2	3.5	${\text{cos}}^{4}$
M_3	20	5	2	4	${\text{cos}}^{4}$
M_4	20	5	2	5	${\text{cos}}^{4}$
M_5	30	0	0	4	sin
M_6	10	0	0	4	sin
M_7	10	0	0	3	sin

A 4D CT study was available for each motion pattern. The phantom in motion was 4D CT scanned in cine mode on a GE LightSpeed 64‐slice CT (General Electric Medical Systems, Waukesha, WI) with 1.5 mm slice thickness. High resolution breathing signal acquired with the real‐time position management system (RMP, Varian Medical Systems, Palo Alto, CA) was used to retrospectively sort the 4D CT dataset into ten 3D CT series that were equally spaced in time.

**Figure 2:**
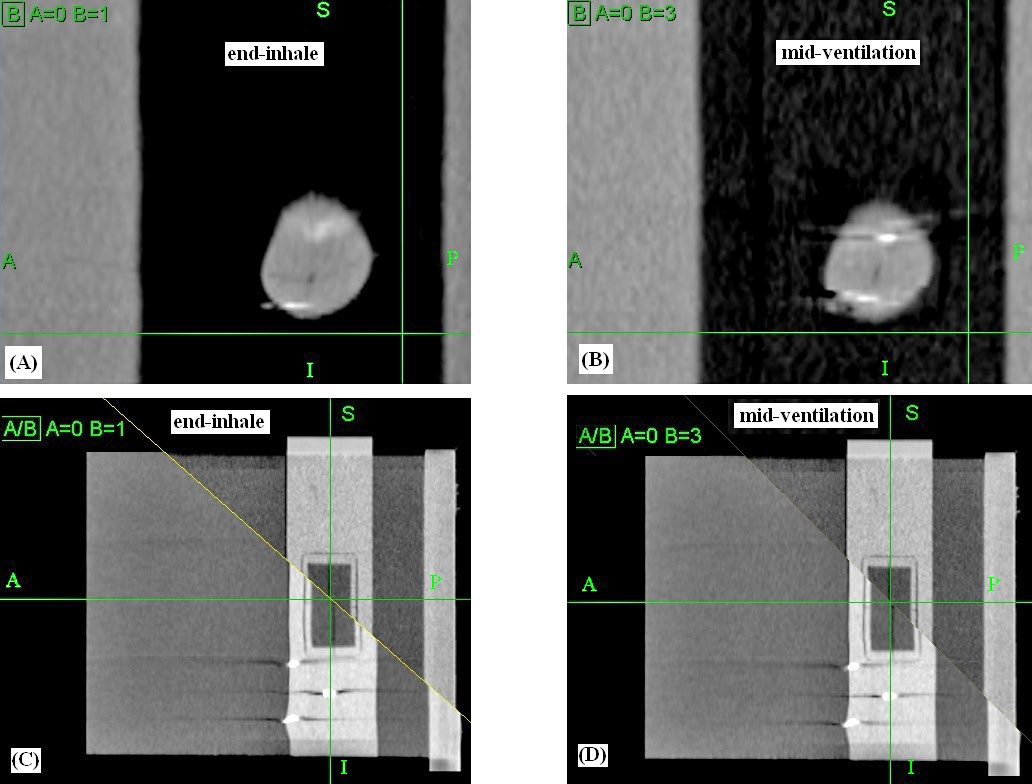
Results of the deformable image registrations (DIR). The deformation yielded apparent distortion of the spherical target ((a) and (b)) and the cord structure ((c) and (d)) in the split view.

Once the ten 3D CT series were reconstructed, they were input to the MultiPlan TPS v.4.0.x (Accuray Inc., Sunnyvale, CA). The 4D dose calculation algorithm implemented in the MultiPlan TPS was described in details by West et al.^(14)^ In brief, it begins with nonrigid image registrations, or deformable image registrations (DIR), between individual 3D CT images in the 4D CT study using a hybrid point‐intensity–based approach. The 4D planning system allowed virtual control points to be placed in regions with low contrast for improving the DIR results. For each 4D CT study, we added a total of ten control points on and near the off‐target measurement films to improve the DIR. The DIR takes as input an anatomical voxel location in the reference 3D CT image and returns as output the deformed position of this anatomical voxel in the other 3D CT images. The resulting deformation vector fields (DVF) are represented by a combination of third‐order B splines. In Fig. 2, the left and right columns show the resulting deformed geometry at the end‐inhale and midventilation phases, respectively. Due to the motion‐related artifacts associated with the 4D CT images, the deformation yielded apparent distortion of the spherical target ((Figs. 2a)and (2b), as well as the neighboring cylindrical cord structure ((Figs. 2c)and (2d).

**Figure 3:**
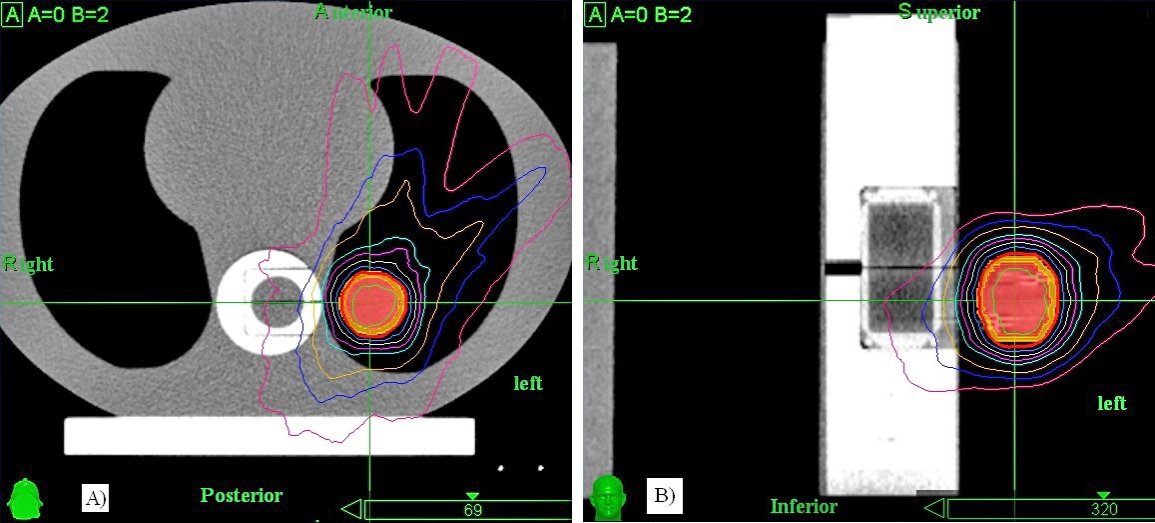
Example of the plan dose distribution showing the geometric relation between the moving target and the static cord structure.

To incorporate the dynamic beam motion and organ deformation, the beam arrangement was shifted according to the positions of the fidudical markers in the 4D CT study, and a standard dose calculation on each of the individual 3D CT images followed. The dose matrix of the reference phase was then deformed to other phase, and doses at each deformed position in these static dose matrices were interpolated back to the reference dose grid of the deformed dose voxel. Finally, the deformed doses were summed and weighted by the relative width of the time bin for each 3D CT frame to compose the 4D dose in the reference CT space image on which structure contours were delineated (Fig. 3) and dose distribution was evaluated. In this study, the target and critical organs, beam geometry, and dose grid were all defined on the reference CT dataset at the end of exhale (EOE) phase.

Based on the 4D dose calculation framework described above, 4D optimization was carried out for each of the motion patterns. The optimization process was similar to standard 3D planning in the way the optimization objectives were specified; the optimization process simply involved substituting the 4D dose matrices for the 3D dose matrices. Optimization objectives were to achieve 99% target coverage, target dose heterogeneity <30%, and minimal monitor units. It should be noted that the target for optimization was 9 mm less in diameter than the actual phantom target in order to fit the target dose distributions to the active film measurement area. The 4D dose distributions were produced with the ray‐tracing algorithm with equivalent path length (EPL) heterogeneity correction(^15^) and 4.2 Gy x three fractions was prescribed to the planning target's surface. The fraction dose was scaled to 4.2 Gy in order to accommodate the dose range applicable to the EBT2 film dosimetry (up to 10 Gy for red channel measurement).

Because of sharp dose falloff, off‐target dose to the cord structure was so low that dose measurements were difficult. Therefore, other sets of 3D optimized plans were created with intention to introduce large dose spillage to the measurement film plane of the cord structure. Subsequently, the optimized 3D beam set was applied to 4D dose calculation. Final 4D dose distributions were calculated for the target and cord plans with the fast Monte Carlo (MC) algorithm at 1% relative uncertainty.^(15)^ Resulting dose distributions were Gaussian smoothed (σ=0.6 %) to reduce statistical noise. The MC dose calculation voxel was approximately $1.05\;\times \;1.05\;\times \;1.5\;{\text{mm}}^{3}$. It is important to note that the electron transport algorithm calculated absorbed dose to variable density water, which is not strictly identical to absorbed dose to medium. However, the difference between these quantities is negligible for most of the biological tissues (including lung and bone), but may be greater in nonbiological materials such as metallic implants.^(16)^

Treatment plans were delivered in two ways. First, the rigid phantom was irradiated according to the treatment plans assuming reproducible target motion during imaging and beam delivery. Second, we evaluated the errors of 4D dose calculation arising from possible systematic variability of target motion by delivering each plan to the phantom moving a motion pattern that is systematically different from the one used in treatment planning. The reason we carried out this evaluation is that, unlike the error of random target motion that has been shown to average out when multiple fractions are delivered, error introduced by systematic change of the target motion pattern in treatment planning and in actual treatments is likely to cause considerable discrepancy between the planned and actual dose distributions.^(12)^ A 3D plan created with MC dose calculation on static 3D images at EOE was also delivered to the phantom in the absence of motion for benchmarking. Table 2 summarizes the dose measurements for different treatment delivery methods.

**Table 2:** Summary of the treatment delivery and the types of motion variability tested.

*Delivered Plan*	*Actual Motion*	*Motion Variability Tested*
M_1/M_3	M_1/M_3	Baseline
M_1	M_3	Increasing amp. (3D motion)
M_3	M_1	Decreasing amp. (3D motion)
M_2/M_4	M_2/M_4	Baseline
M_4	M_2	Increasing freq. (3D motion)
M_2	M_4	Decreasing freq. (3D motion)
M_6/M_7	M_6/M_7	Baseline
M_6	M_7	Increasing freq. (1D motion)^a^
M_7	M_6	Decreasing freq. (1D motion)^a^
M_5/M_7	M_5/M_7	Baseline
M_5	M_7	Decreasing amp.+increasing freq. (1D motion)^a^
M_7	M_5	Increasing amp.+decreasing freq. (1D motion)^a^
M_1/M_6	M_6/M_1	Baseline
M_1	M_6	Decreasing amp. (3D vs. 1D motion) + waveform (${\text{cos}}^{4}$ vs. *sin*)
M_6	M_1	Increasing amp. (1D vs. 3D motion) + waveform (*sin* vs. ${\text{cos}}^{4}$)
M_5/M_6	M_5/M_6	Baseline
M_5	M_6	Decreasing amp. (1D motion)^a,b^
M_6	M_5	Increasing amp. (1D motion)^a,b^

^a^Tested for off‐target films; ^b^not tested for target films.

GAFCHROMIC EBT2 films (International Specialty Products, Wayne, NJ) were used for dose measurements because of its high spatial resolution, minimal angular and energy response compared to other common dosimetry tools such as ionization chamber and thermoluminescence dosimeters.^17^, ^18^ Dose measurements were performed separately for the coronal and transverse films in the target, so for each motion pattern the same plan was delivered six times. Off‐target dose measurements of the cord structure were made only for linear motions (M_5 to M_7). The exposed films were scanned within 24 hours postirradiation on an Epson Expression 1000XL flatbed scanner (Epson America, Long Beach, CA) to produce a 48 bit color (RGB) images of 150 dots per inch resolution. Each exposed film was scanned three times and averaged to reduce the scanner electronic noise. No marker‐dye correction for the nonuniformity of EBT2 film was made for the calibration films and measurement films because of the limited dose sensitivity of blue channel images above 4 Gy.^(19)^ At lower dose range, Richley et al.^(20)^ have demonstrated that intra‐ and intersheet inhomogeneity of film thickness contributed to 2.4% dose uncertainty at 200 cGy. Absolute EBT2 film dosimetry without applying blue channel corrections have been reported to show good results in quality assurance of intensity‐modulated radiotherapy.^(18)^ The red channel images were registered to the dose plane exported from the TPS at the EOE phase with the FilmQA software (3cognition LLC. Wayne, NJ) and were analyzed based on the γ metric. The films were compared to the calculated dose based on the γ metric, which aims to quantitatively compare the measured and calculated dose distributions by dose differences and distance to agreement (DTA). In principle, the γ criteria should be set in accordance to desired dosimetric accuracy of the treatments. Considering the dose uncertainty of EBT2 film dosimetry and the increased sensitivity of measurement errors to the large dose gradient of SBRT, the passing criteria were set at 5% of the absolute dose difference and 3 mm DTA (5%/3 mm) assuming 1 mm error in residual tracking accuracy and 2 mm error associated with the deformable image registration and film alignment during measurement and analysis. The 5%/3 mm criteria was less stringent than the 3%/3 mm criteria recommended in the AAPM Report 135 for quality assurance of robotic radiosurgery,^(21)^ but it was deemed acceptable for heterogeneous phantom by the Radiological Physics Center (RPC) for National Cancer Institute clinical trials. We further considered the acceptance level of percentage of pixels passing the 5%/3 mm to be ≤90%. We compared the percentages of pixels passing among the various motion patterns (sin vs. $\cos^{4}$), and with and without systematic change of motion patterns (e.g., reproducible motion vs. increased and decreased amplitude), using Mann‐Whitney U tests. All tests were considered significant at p < 0.05.

## III. RESULTS

**Figure 4:**
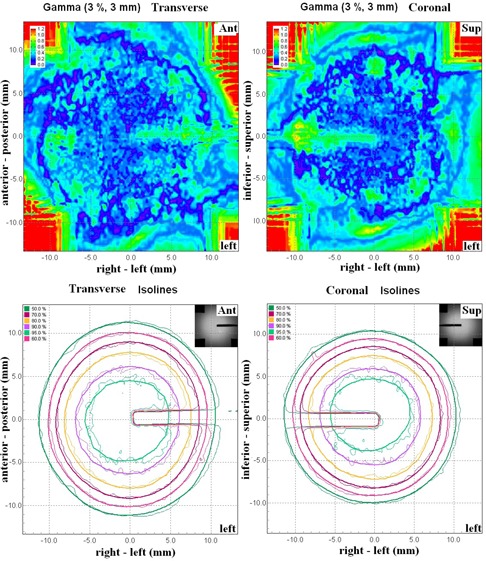
Gamma (3%/3 mm) distributions (top row), overlaid measured (thin solid line), calculated (thick solid line) and isodose lines (bottom row) in the transverse and coronal films of the benchmark static Monte Carlo dose plan.

**Figure 5:**
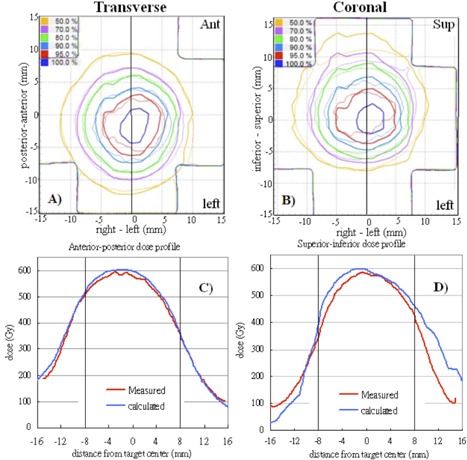
Overlaid measured (thin solid line) and calculated (thick solid line) dose distributions in the transverse film (a) and coronal film (b) are showed for the same 4D plan in Calculated and measured dose profiles corresponding to the solid lines in (a) and (b) are plotted in (c) and (d). The dashed lines represent the target region. Measured dose=thin solid lines; calculated dose=thick solid lines.

Table 3 shows the mean percentages of pixels passing the 5%/3 mm γ criteria with the film measurements for benchmark static plan and reproducible motion plans. For the benchmark 3D MC plan, all pixels evaluated passed at the tighter 3%/3 mm γ criteria as a consequence of excellent agreement between the measured and calculated dose distributions (Fig. 4). Overall, the percentages passing for the 4D MC plans were 95% ±7% (mean±one standard deviation (SD)). Out of 42 films, the percentage of pixels passing was smaller than 90% in eight films. In the failing films, the average percentages of pixels passing were 85% for six coronal films, and 77% for two transverse films, respectively. No significant difference was observed between 1D sin and 3D ${\text{cos}}^{4}$ waveforms (p=0.27). Figure 5 shows the overlaid measured and calculated relative isodose lines in the transverse and coronal planes for a 3D ${\text{cos}}^{4}$ motion (M_3). Although these film planes satisfied the acceptance criteria, the overlaid isodose lines exhibited some regions of disagreement, particularly in the coronal plane where misalignment of the isodose lines deteriorated from inferior to superior direction (i.e., from exhalation to inhalation) ((Figs. 5a)and (5b). (Fig. 5c)and (5d) clearly indicated that the 4D plan reproduced the dose profile fairly well in the anterior–posterior motion axis, but failed to model the dose profile in the principal motion axis. It stretched the dose profile to superior (end‐exhale) and compressed the dose profile to inferior (end‐inhale). This trend was observed mainly in 4D plans of ${\text{cos}}^{4}$ motion.

**Table 3:** Percentage of pixels passing the 5%/3 mm criteria in the moving target and cord cylinder for various motion patterns and dose calculation methods.

*Motion Pattern*	*Film Orientation*	*Percentages of Pixels Passing 5%/3 mm*
Target		
M_1	Coronal	94±8
	Transverse	89±12
	Mean±SD	92±5
M_2	Coronal	91±8
	Transverse	92±10
	Mean±SD	91±4
M_3	Coronal	95±7
	Transverse	100±0
	Mean±SD	98±4
M_4	Coronal	94±7
	Transverse	99±1
	Mean±SD	96±3
M_5	Coronal	99±1
	Transverse	100±0
	Mean±SD	99±0
M_6	Coronal	96±6
	Transverse	100±0
	Mean±SD	97±3
M_7	Coronal	86±6
	Transverse	97±5
	Mean±SD	91±1
Overall	Mean±SD	95±7
Off‐target OAR		
M_5	Coronal	99±2
M_6	Coronal	88±8
M_7	Coronal	85±15
Overall	Mean±SD	91±10

**Figure 6:**
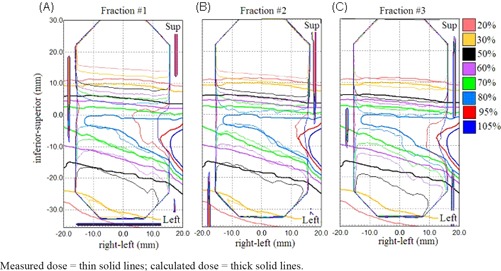
Dose distributions measured when the same treatment plan for motion pattern M_7 was delivered three times. Random relationship between respiratory phase and beam‐on time resulted in slightly different dose distributions.

For off‐target structure, three films were failed and the mean percentage of pixels passing was 91% ±11%. Separate evaluations of the dose difference maps and the distance‐to‐agreement maps demonstrated that failing regions were mainly attributed to dose difference. Figure 6 shows the measured dose distributions of the same treatment plan. Taking out the uncertainty of experiment setup, the slight difference between these dose distributions was presumably a result of the random relationship between breathing phase and beam‐on time.

**Figure 7:**
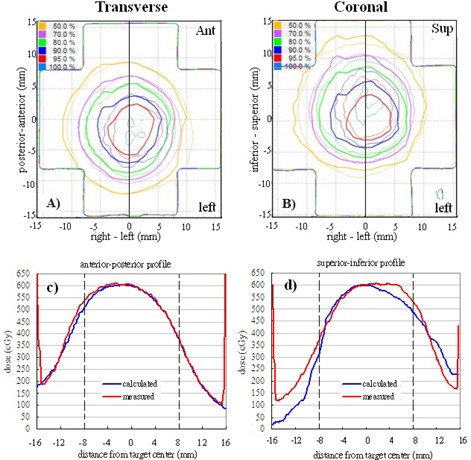
Overlaid calculated (thick solid line) and measured (thin solid line) dose distributions in the axial (left column) and coronal (right column) target films are shown for treatment plan that was created based on motion pattern M_3 and was intentionally delivered to the phantom moving in motion pattern M_1. Calculated and measured dose profiles corresponding to the solid lines in (a) and (b) are plotted in (c) and (d). The dashed lines represent the target region.

**Figure 8:**
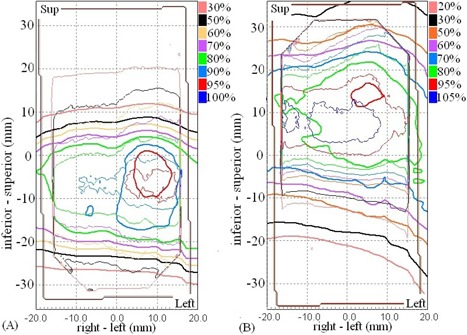
Measured dose distributions (thin solid line) with 4D plans (thick solid line) delivered in the presence of systematic increase of motion amplitude (a) and systematic decrease of motion amplitude (b) during treatment deliveries.

The measurement results for irreproducible target motion were summarized in Table 4. The results in the target and the off‐target cord were different. For the target, the average passing rates were 92% ±6%. Out of 60 films, the percentages of pixels passing were smaller than 90% in ten coronal films (82% ±7%) and seven axial films (77% ±9%). The γ passing rate was not significantly different between reproducible and irreproducible target motions (p=0.324). Also, the γ passing rate did not show any significant change for each type of motion variation compared to the results obtained with the corresponding plan that was delivered to reproducible motion. As a comparison to the dose distributions of reproducible motion shown in Fig. 5, Fig. 7 shows the overlaid calculated and measured dose distribution for treatment plan that was created with the same motion pattern as in Fig. 5 (M_3) and was intentionally delivered to the phantom moving in a different motion pattern (M_1) of reduced motion range. Similarly, the calculated dose distribution and profile demonstrated greater discrepancies with the measured dose distribution inside and outside the target area in the coronal film than in the axial film. The effect of reduced motion range was that the delivered dose was concentrated to a smaller region of higher dose inside the target ((Fig. 7b)and (7d). On the other hand, the systematic change of motion pattern strongly affected the doses to the off‐target structures, as all measurements had percentages of pixels passing well below 90% with a mean of 48% ±14%. The γ passing rate was significantly lower in irreproducible motions than in reproducible motions (p < 0.05). As illustrated in Fig. 8, the effect of increased and decreased motion amplitude was mirrored. The increased motion amplitude spread out the off‐target dose distribution, decreasing the region of high dose and increasing the region of low dose ((Fig. 8a). The decreased motion amplitude squeezed the isodose lines, increasing the high‐dose region while decreasing the low‐dose region ((Fig. 8b). In this case, the 50% and 30% isodose lines were shifted by a maximum distance of 6.5 mm and 8.7 mm, respectively. Of more importance is that the maximum dose was increased by more than 10% compared to the plan estimate.

**Table 4:** Percentages of pixels passing 5%/3 mm gamma criteria with introduction of various systematic changes of motion pattern.

*Delivered Plan*	*Actual Motion*	*Motion Variability*	*Percentages of Pixels Passing* 5%/3 mm *in Coronal Films*	*Percentages of Pixel Passing* 5%/3 mm *in Axial Films*
Target				
M_1	M_3	Amp. (3D motion)	97±3	99±2
M_3	M_1	Amp. (3D motion)	94±6	99±2
M_2	M_4	Freq. (3D motion)	92±9	84±7
M_4	M_2	Freq. (3D motion)	98±2	74±16
M_6	M_7	Freq. (1D motion)	92±6	99±1
M_7	M_6	Freq. (1D motion)	91±9	100±0
M_5	M_7	Amp.+Freq. (1D motion)	96±5	96±6
M_7	M_5	Amp.+Freq. (1D motion)	81±14	92±10
M_1	M_6	Amp.+Waveform (3D ${\text{cos}}^{4}$ vs.1D sin)	90±8	100±0
M_6	M_1	Amp.+Waveform (3D ${\text{cos}}^{4}$ vs.1D sin)	84±9	97±3
Off‐target Cord				
M_5	M_6	Amp. (1D motion)	59±2	
M_6	M_5	Amp. (1D motion)	46±15	
M_6	M_7	Freq. (1D motion)	35±4	
M_7	M_6	Freq. (1D motion)	48±7	
M_7	M_5	Amp.+Freq. (1D motion)	34±15	
M_5	M_7	Amp.+Freq. (1D motion)	62±10	

## IV. DISCUSSION

4D dose calculation has been used to explicitly account for the dose blurring effect of intrafractional target motion. Previous studies have evaluated and compared 3D and 4D dose calculations with measured target doses using rigid and deformable lung phantoms in conventional radiotherapy.^6^, ^22^ This work aimed to evaluate the accuracy of a commercial 4D dose calculation algorithm for CyberKnife.

For the moving target, the mean percentages of pixels passing were 95% when the target motion was consistent between treatment planning and delivery. Introduction of systematic increasing and/or decreasing amplitude and frequency during actual treatments did not lead to significant differences in the passing rates (p < 0.05). These results were not surprising. Previously, Niousitkou et al.^(23)^ showed 97% pixels passing at 3%/3 mm γ criteria, even if treatment plans were created for deformable lung phantom using 3D dose calculation and simple equivalent path length correction for heterogeneity. With real‐time tumor tracking of target, the dose blurring due to respiratory motion is minimized because the target itself did not appear to be moving while it was being tracked by the treatment beams. Conversely, off‐target structures, such as spinal cord that did not move in the same way with the tracking beams, appeared to be moving and dose blurring was primarily manifested on these structures. As shown in Fig. 6, 4D dose calculation algorithm adequately predicted the dose distributions in the off‐target cord structure in reproducible motions, and six of the nine measured off‐target films satisfied 90% pixels passing at 5%/3 mm with a mean passing rate of 91%. Nonetheless, 4D dose calculation algorithm apparently failed to predict the dose distribution when the target motion was not the same as captured in the 4D CT dataset. In such cases, all off‐target measured films failed, and the mean percentage of pixels passing reduced significantly to 48%. In order to make 85%–90% of pixels passing the evaluations, the γ criteria had to be relaxed to 10%/5 mm and even 15%/7 mm. It clearly suggested that the sensitivity of 4D dose calculations to variation of target motion differs by structures of interest. It should be noted that our results were based on rigid geometry and rigid target motion. However, errors due to differential target motion and varying lung density should be negligible if adequate safety margin is used.^5^, ^24^ Errors due to variation of beam paths as treatment beams traversing through different materials were also expected to averaged out for a large number of beams (>100 beams) used in Synchrony treatment.

One of the difficulties to verify 4D dose distributions was the selection of the calculated dose plane for comparisons with measured films. From Fig. 5, dose plane exported using the centroid position of the target at the end‐exhale led to a systematic shift from the measured dose distributions because the end‐exhale phase did not correspond to the time‐weighted average target position. Furthermore, the time‐weighted average position, as portrayed on the 4D CT data, may not necessarily correspond to the same geometrical point if the motion pattern has changed in the actual treatment. Such discrepancy between the exported dose plane and the measured film plan added to the uncertainty of the evaluated accuracy of the 4D dose calculations. Indeed, it would not be possible to outline the target and to define the dose plane at the time‐weighted average position or midventilation phase because of significant motion artifacts associated with these reconstructed 4D CT images. For the same reason, we avoided using the time‐weighted average images as the reference frame to register with other CT frames because it would affect all the calculated DVFs. Similarly, the distorted medium density maps, when applied to determine the energy lost, step length, and step direction in the particle transport processes, were expected to contribute to significant error of the resulting dose distributions. For instance, the increased CT number caused by the distortion of metal markers ((Fig. 2b) may cause the electrons to terminate their tracks and deposit their energies that would be otherwise carried away from the spot. On the other hand, the approximate efficiency improvement techniques implemented in the high‐energy inelastic interaction calculation for electron transport, which may cause substantial difference between absorbed dose to variable density water in the MultiPlan Monte Carlo algorithm and absorbed dose to medium,^(25)^ were expected to exaggerate errors of the dose calculation results given the significant distortion of the high‐density metal markers in the CT images. The errors of DIRs and dose calculations due to image artifacts can be clearly visualized by comparing the dose distribution calculated on static geometry (Fig. 4) and on temporally changing geometry (Fig. 5). As noted in Fig. 5, the artifacts were primarily manifested in the flexuous isodose lines in the coronal plane which gradually became smooth to the inferior of the dose distribution (i.e., in the exhalation direction). The better agreement between the calculated and measured dose distributions to inferior can be explained by the improved accuracy of the DIRs and dose calculations.

According to West et al.,^(14)^ the 4D dose calculation algorithm distributed the dose or MU of each beam to all breathing phases according to their relative weights. That is, the dose (MU) of each beam was phase‐locked. But when the beam was started at any random breathing phase, the dose and MU was redistributed randomly. In the worst scenario, some phases that would have received some doses would end up receiving no doses before the beam had delivered all MUs. However, this situation is unlikely because SBRT usually delivers up to 20 Gy per fraction and the duration of each beam shall be comparable to breathing cycle. Even if the fractional dose of this study was scaled down by a factor of 4.5 as compared to the actual treatment dose, the effect of random relationship between the breathing phase and the beam‐on time was negligible. It led to slight isodose misalignments between measurements, as shown in the off‐target films (Fig. 6). This work focused on evaluating the 4D dose calculation algorithm of the CyberKnife TPS. We did not compare the accuracy of 3D and 4D dose calculations. In theory, 4D dose optimization and calculation should provide better target dose coverage by taking explicit account of the organ deformation and motion. The inclusion of beam movements in the calculation should also improve the estimated doses to off‐target critical structures. 4D dose calculation requires interpolations of dose matrices between multiple geometries. Few studies have suggested that the interpolation error is more pronounced in high‐dose gradients.^9^, ^26^ This may have some implication in SBRT treatment because treatment dose is often prescribed to lower isodose line producing highly heterogeneous dose distributions. The disagreement between the calculated and measured dose profile observed in this study may also be a consequence of interpolation errors (Fig. 5). It is possible to reduce the interpolation error by using smaller dose calculation grid at the expense of increased calculation time, which is already ten times more than standard 3D dose calculation.^(27)^ Nevertheless, there are still inherent interpolation errors that cannot be reduced by using smaller dose grid without considering the conservation of mass and energy in the dose mapping process.^(10)^ On the other hand, actual organ motion and deformation is generally not known a priori, and the planning 4D CT contains motion information at a specific time point. Interfractional and intrafractional variations of target motion range, period, and volume are frequently noted in lung radiotherapy.^1^, ^28^ Systematic and random variation of target motion results in shifting and blurring off‐target dose distributions and thus narrowing the advantages of 4D dose calculation to better predict the off‐target doses. That 4D dose optimization and calculation can facilitate further margin reduction is therefore questionable. On the other hand, the planning risk volume (PRV) has been recommended by ICRU Report 83 for protection of organs‐at‐risk from overdosed.^(29)^ Although studied data of positional variations of these organs can serve as the baseline for setting up the PRVs, Stroom and Heijmen^(30)^ have demonstrated the limitation of PRV with organs that are organized in serial function units because of the nonlinear behavior of these organs with dose gradient. As indicated in (Fig. 8b), a systematic decrease of target motion concentrated the delivered dose to a smaller zone of the cord structure and increased the maximum dose by <10%, compared to the plan estimate. This may have great impacts on the simulated cord structure that is organized in serial function units, particularly when the original planned maximum dose has already been touching the organ tolerance dose. This problem cannot be simply resolved by adding a PRV to the cord structure. To make 4D planning of real‐time tumor tracking robust against uncertainty of the virtual patient model, robust optimization proposed by Heath et al.^(31)^ should be incorporated in the current 4D treatment planning module in the CyberKnife TPS.

## V. CONCLUSIONS

This work demonstrated that the commercial 4D dose calculation algorithm for real‐time tumor tracking SBRT was able to account for the dynamic beam movement and provided adequate predictions of the doses to moving target, as well as static off‐target structure with reproducible target motion. However, large discrepancies between the 4D calculated and measured dose distributions, particularly in the off‐target structure, were observed when systematic change of motion pattern occurred during beam delivery. These results suggest that the accuracy of 4D dose calculation, particularly in off‐target stationary structure, is strongly tied to the regularity of target motion, and that the solutions of 4D planning do not reflect the clinical nature of nonreproducible target motion generally.

## ACKNOWLEDGMENTS

This work was supported by Hong Kong Adventist Hospital.
